# A randomized crossover trial to assess therapeutic efficacy and cost reduction of acid ursodeoxycholic manufactured by the university hospital for the treatment of primary biliary cholangitis

**DOI:** 10.1186/s12876-020-01399-5

**Published:** 2020-08-05

**Authors:** Larissa Akeme Nakano, Eduardo Luiz Rachid Cançado, Cleuber Esteves Chaves, Maria Cristina Vaz Madeira, Jéssica Toshie Katayose, Mariana Akemi Nabeshima, Victor Fossaluza, Gabriela Guimarães Uhrigshardt, Zheng Liting, Vanusa Barbosa Pinto, Flair José Carrilho, Suzane Kioko Ono

**Affiliations:** 1 Department of Gastroenterology, Division of Clinical Gastroenterology and Hepatology, Hospital das Clinicas, University of São Paulo School of Medicine, Av. Dr. Enéas Carvalho de Aguiar, 255, ICHC, 9th Floor, office 9159, São Paulo, SP 05403-000 Brazil; 2 Laboratory of Medical Investigation LIM 06, Institute of Tropical Medicine, University of São Paulo, São Paulo, Brazil; 3 Division of Pharmacy of Hospital das Clínicas, University of São Paulo School of Medicine, São Paulo, Brazil; 4 Institute of Mathematics and Statistics, University of São Paulo, São Paulo, Brazil

**Keywords:** Ursodeoxycholic acid, Primary biliary cholangitis, Capsules, Tablets, Health care costs, Hospital

## Abstract

**Background:**

Health care costs are growing faster than the rest of the global economy, according to the World Health Organization (WHO). Countries’ health expenditures include paying for general medicine, diagnostic procedures, hospitalizations and surgeries, as well as medications and prescribed treatment. Primary biliary cholangitis (PBC) is a rare autoimmune liver disease and the first line available treatment is ursodeoxycholic acid (UDCA), however, direct and indirect treatment costs are expensive. Main aim of this trial was to assess if the therapeutic efficacy of UDCA manufactured by the university hospital is equivalent to that of standard UDCA and treatment cost reduction in patients with PBC.

**Methods:**

It is a prospective, interventional, randomized, and crossover study in patients diagnosed with PBC. UDCA 300 mg tablets and capsules were developed and manufactured by the university hospital. Thirty patients under treatment with standard UDCA, in stable doses were randomized in sequence A and B, 15 patients in each arm. The groups were treated for 12 weeks and after, the UDCA formulation was changed, following for another 12 weeks of continuous therapy (tablets and capsules / capsules and tablets). Laboratory tests were performed at time T0 (beginning of treatment), T1 (at the 12 week-therapy, before the crossing-over) and T2 (end of treatment). The evaluation was done by comparing the hepatic parameters ALP, GGT, ALT, AST and total bilirubin, also considering the adverse events. The comparison of costs was based on price of the manufactured UDCA and standard UDCA price of the hospital.

**Results:**

Hospital reduced 66.1% the PBC treatment costs using manufactured UDCA. There were no differences in the biochemical parameters between sequence (A and B) and tablets or capsules of UDCA formulations applied in the treatment of PBC.

**Conclusions:**

The study showed that there was no significant difference between manufactured UDCA (capsule and tablet) and standard UDCA. Hospital reduced the PBC treatment costs using the manufactured UDCA by the university hospital.

**Trial registration:**

ClinicalTrials.gov: NCT03489889 retrospectively registered on January 12th, 2018; Ethics Committee approved the study (ID: 1.790.088) on October 25th, 2016.

## Background

Costs associated with health care services are growing. Health systems and providers need to consider the effectiveness and economic impact of existing service models, and determine if there are alternatives that might lead to improved efficiencies without compromising the quality of care and patient outcomes [1, 2].

Drugs have a major impact on increasing hospital costs. The main objective is to provide effective and safe pharmacotherapy at the lowest possible cost [2]. Pharmacoeconomics is an important strategy for therapeutic rationalization, allowing evaluating different variables such as cost, effectiveness, benefit, utility and efficiency of different treatments [3].

The manufactured drug in hospital is a cost-effective alternative. The pharmacy produces drugs with similar to commercially distributed products, exclusive formulations for routine consumption, and dealt with special demands related to clinical trials [4].

Standard ursodeoxycholic acid is a high-cost drug and it is the third drug with greater financial impact at Hospital das Clínicas. Reduction of costs with the use of manufactured UDCA by the university hospital was an alternative to assure the patients’ treatment.

Ursodeoxycholic acid is the first-line pharmacotherapy for primary biliary cholangitis (PBC) [5, 6]. It is a chronic, autoimmune liver disease characterized by progressive cholestasis, eventually leading to cirrhosis [5, 6]. Affects predominantly women from 30 to 65 years old of age [5, 7]. The incidence and prevalence rates range from 0.33–5.80 per 100,000 and 1.91–40.20 per 100,000 inhabitants/year, respectively [8].

Aim of this trial was to assess if the therapeutic efficacy of UDCA manufactured by the university hospital is equivalent to that standard UDCA and treatment’ cost reduction in patients with PBC. The secondary objectives were to assess the preference of drug formulations.

## Methods

### Trial design

The trial was an interventional, prospective, randomized, crossover design of 24 weeks’ duration. It was conducted at the Hospital das Clínicas da Faculdade de Medicina da Universidade de São Paulo located in the city of São Paulo, Brazil.

A crossover design was chosen for this study instead of the more traditional randomized, parallel-group design because the within-patient variation is less than the between patient variation and thus required fewer patients. We did not include a washout period between treatments to increase patient safety [9–11].

This trial used the extensions to randomized crossover trials of the CONSORT (Consolidated Standards of Reporting Trials) 2010 Statement [12].

### Participants

Patients with PBC attending at Hospital das Clínicas between December 2016 and May 2018 were screened. The diagnosis of PBC was made according to European Association for the Study of the Liver (EASL) and American Association for the Study of the Liver Disease (AASLD) guidelines, when at least two of the following three criteria were fulfilled: a) biochemical evidence of cholestasis based on alkaline phosphatase (ALP) elevation, more than 1.5 times upper limits normal (ULN), b) antimitochondrial antibodies (AMA) reactivity, or other PBC-specific auto-antibodies, including antinuclear antibodies with specificity to sp100 or gp210, if AMA is negative, or c) histologic findings of nonsuppurative destructive cholangitis and destruction of interlobular bile ducts [5, 6].

### Inclusion criteria

Male or female patients with PBC, ≥18 years old that provided written informed consent were considered eligible for the study. These patients must have been under treatment with the reference pharmaceutical product (referred as standard UDCA) for at least 6 months before starting the hospital formulation. The dose of the UDCA manufactured by the university hospital for each patient was the same she/he was using previously.

### Exclusion criteria

The presence of any other co-existing liver diseases, pregnancy, known intolerance to the study drugs formula or if the patients did not provide the written informed consent.

### Investigational product

Hospital pharmacy was responsible for manufactured two different formulations, UDCA 300 mg tablets and capsules in accordance with the good manufacturing practice regulations. The quality control of the material based on the British Pharmacopoeia 2007, which provided the official standards for pharmaceutical substances.

### Randomization

Eligible subjects were randomized in a 1:1 allocation to one of two treatment sequences - UDCA 300 mg tablets/ capsules or UDCA 300 mg capsules/ tablets - and received each treatment for 12 weeks. A simple randomization numbers list was generated using “RANDBETWEEN” the Microsoft Office Excel.

### Interventions

All patients received initial treatment with standard UDCA for at least 6 months before starting the trial. The individual dose of UDCA was accordingly body weight (13 to 15 mg/kg/day). There were two consecutive sequences (A and B). Within 24 weeks, six visits were performed. Patients in the sequence A were treated with UDCA 300 mg tablets for the first 12 weeks followed by UDCA 300 mg capsules for 12 weeks. In sequence B, patients received UDCA 300 mg capsules for the first 12 weeks followed by UDCA 300 mg tablets for 12 weeks. It was a 2 × 2 randomised crossover trial and the intention is that all participants receive both of the interventions.

### Study endpoints

The primary outcome was to determine if the therapeutic efficacy of UDCA 300 mg tablets and capsules manufactured by the university hospital is equivalent to that of standard UDCA 300 mg in patients with PBC. This was assessed by comparing the liver enzyme parameters: alkaline phosphatase (ALP), gamma glutamyl transferase (GGT), alanine aminotransferase (ALT), aspartate aminotransferase (AST), and total bilirubin (TBIL) after used the standard UDCA 300 mg (Time 0), end of the first treatment period with UDCA 300 mg capsules/tablets (Time 1) and the end of the last treatment period with UDCA 300 mg tablets/capsules (Time 2).

The laboratory parameters were measured in a hospital laboratory. The therapeutic efficacy was defined as liver enzyme parameters within the normal reference range and ALP below 1.5x the normal upper reference value. Secondary outcomes were to assess the treatment cost reduction in patients with PBC and preference of drug formulations. They was assessed at the termination visit.

### Monitoring and follow-up

The patients were monitored clinically and underwent routine tests as described. They were regularly informed and advised for regular medication intake. The adherence was checked through the counting of the number of tablets/capsules left in the bottle at each visit.

### Treatment’ costs

Treatment’ costs were assessed by comparing the price of UDCA manufactured by the university hospital and standard UDCA. The trial considered all the period of the treatment, 12 weeks with capsule and 12 weeks with tablets.

### Sample size and statistical methods

The initial sample size calculations were performed using a power of 80% was targeted, the overall one-sided level of significance was 0.025, the non-inferiority margin was 15% absolute, and a standard deviation of 25% was assumed. Based on the interim analysis it was then planned to randomize a total of 64 patients.

The main hypothesis of therapeutic equivalence consisted of no difference between UDCA manufactured by the university hospital and standard UDCA. In addition, a low cost treatment using manufactured UDCA. Statistical techniques used were descriptive one-dimensional analysis and descriptive analysis multidimensional, and inferential analysis considering an ANOVA test and Wald test. A *p*-value < 0.05 was considered statistically significant, refusing the null hypothesis. The program used for all calculations was R version 3.3.2 and RStudio version 1.0.143.

## Results

**Fig. 1:**
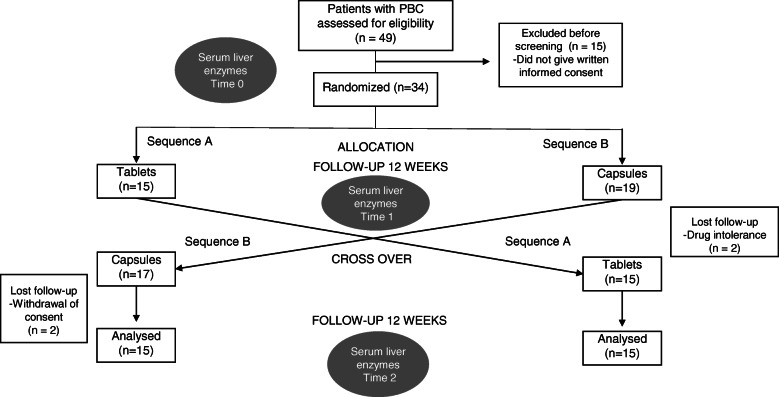
CONSORT diagram of patients’ recruitment and analysis. Study design and randomization: Thirty patients under treatment with commercial UDCA, in stable doses were randomized in groups A and B, 15 patients in each arm. The groups were treated for 12 weeks and after, the UDCA formulation was changed, following for another 12 weeks of continuous therapy (tablets and capsules / capsules and tablets). Laboratory tests were performed at time T0 (beginning of treatment), T1 (at the 12 week-therapy, before the crossing-over) and T2 (end of treatment)

Initially, 49 patients were screened, and 15 were excluded because they did not provide written informed consent. Therefore, 34 patients were recruited and randomized into the study (Fig. 1). After randomization, four patients left the trial prematurely (withdrawal of consent) and were therefore not included in any statistical analysis, just adverse event.

### Baseline demographic characteristics, clinical parameters and laboratory data

Baseline characteristics of the 30 patients are summarized in Table 1. As expected, most patients were female (93.33%) and caucasian race (66.67%). The mean age at baseline was 56.73 ± 11.48 years old, and the age range was between 33 and 72 years old. The most frequent symptoms at baseline were pruritus (60%). At baseline, the mean AL*P* value was 126.10 U/L and the mean GGT value 105.73 U/L, both higher than the upper limit normal. The majority of patients had histologic test (80%). The mean dose of the drug was 13.89 ± 1.76 mg/kg/day, which was in accordance with the recommendations by the AASLD and EASL guidelines (13–15 mg/kg/day) [5, 6].

**Table 1:** Baseline characteristics of patients with primary biliary cholangitis. Baseline characteristics of 30 patients with primary biliary cholangitis including gender, age at baseline, time since diagnosis, laboratory parameters (ALP, GGT, ALT, AST and TBIL), stage of PBC (at time of diagnosis, according to histology), symptoms of PBC and drug dose per body weight (mg/kg/d)

Baseline characteristics		Total (*n* = 30)
Gender		
Female	n (%)	28 (93.33)
Male	n (%)	2 (6.67)
Age at baseline (years)	Mean ± SD	56.73 ± 11.48
Time since diagnosis, (years)	Mean ± SD	10.77 ± 5.26
Laboratory parameters		
ALP (U/L)	Mean ± SD	126.10 ± 91.13
GGT (U/L)	Mean ± SD	105.73 ± 118.22
ALT (U/L)	Mean ± SD	35.78 ± 26.54
AST (U/L)	Mean ± SD	33.92 ± 18.10
TBIL (mg/dL)	Mean ± SD	0.59 ± 0.31
Stage of PBC (at time of diagnosis, according to histology)		
Stage I	n (%)	4 (13.33)
Stage II	n (%)	4 (13.33)
Stage III or IV	n (%)	5 (16.67)
Unknown stage	n (%)	11 (36.67)
No histology	n (%)	6 (20)
Symptoms of PBC		
Pruritus	n (%)	18 (60)
Asymptomatic	n (%)	7 (23.33)
Pruritus and fatigue	n (%)	5 (16.67)
Drug dose per body weight (mg/kg/d)	Mean ± SD	13.89 ± 1.76

*SD* standard deviation, *PBC* primary biliary cholangitis, *ALP* alkaline phosphatase, *ALT* alanine aminotransferase, *AST* aspartate aminotransferase, *GGT* gamma glutamyl transpeptidase, *TBIL* total bilirubin

### Biochemical responses

Table 2 shows the changes in serum liver tests during the administration of sequences A and B in three different times. The lack of significance in the effect indicated that there were no changes in the behavior of the variable “response” throughout the study for each of the groups, that is, in all drug exchanges UDCA manufactured by the university hospital, and standard UDCA capsules and tablets, there were no significant changes in the test results, indicating that there are no differences between the drugs.

**Table 2:** Changes in serum liver test during the administration of sequences A and B in three different times. Comparation in serum liver tests during the administration of sequences A and B in three different times (T0, T1 and T2). There were no significant changes in the test results, indicating that there are no differences between the drugs. These results showed the therapeutic efficacy of the drug manufactured in capsules and tablets relative to the standard drug.

Laboratory parameters	Sequence A Tablets= > Capsules (*n* = 15)			Sequence B Capsules= > Tablets (*n* = 15)			*P* value
	T0	T1	T2	T0	T1	T2	
Alkaline phosphatase (U/L)	155.20 ± 120.03	149.30 ± 90.83	156.40 ± 110.15	97.00 ± 31.31	105.27 ± 49.06	101.20 ± 31.79	0.888
Gamma glutamyl transferase (U/L)	95.13 ± 126.14	95.47 ± 121.81	96.07 ± 120.11	116.33 ± 113.13	136.67 ± 162.93	117.33 ± 118.11	0.579
Alanine aminotransferase (U/L)	32.00 ± 31.33	33.93 ± 39.06	35.13 ± 40.23	39.57 ± 21.15	35.07 ± 17.94	53.53 ± 75.89	0.579
Aspartate aminotransferase (U/L)	31.33 ± 21.07	31.93 ± 22.75	32.87 ± 21.55	36.51 ± 14.85	34.53 ± 10.45	46.53 ± 44.89	0.917
Total bilirubin (mg/dL)	0.60 ± 0.33	0.63 ± 0.31	0.62 ± 0.35	0.60 ± 0.31	0.58 ± 0.22	0.56 ± 0.21	0.590

### Treatment’ costs

The treatment’ costs represent the total value of US$7910.95 for 30 patients that used UDCA 300 mg capsules and tablets manufactured by the university hospital during all 24 weeks period. Using standard UDCA and considering the price for a public hospital, the costs of treatment was US$23,358.25 (Table 3). This difference between treatment costs was US$ 15,447.35 (66.1% reduction).

**Table 3:** Treatment costs (*n* = 30). The treatment’ costs represents the total value of US$7910.95 for 30 patients that used UDCA 300 mg capsules and tablets manufactured by the university hospital during all 24 weeks period. Using standard UDCA and considering the price for a public hospital, the costs of treatment was US$23,358.25

	Standard UDCA (24 weeks)	UDCA capsules manufactured (12 weeks)	UDCA tablets manufactured (12 weeks)	UDCA manufactured (24 weeks)
Total (US$)	23,358.25	3988.53	3922.42	7910.95
Mean/patient (US$)	778.61	132.95	130.75	263.70

*UDCA* acid ursodeoxycholic

### The patients’ preference of study medication

**Fig. 2:**
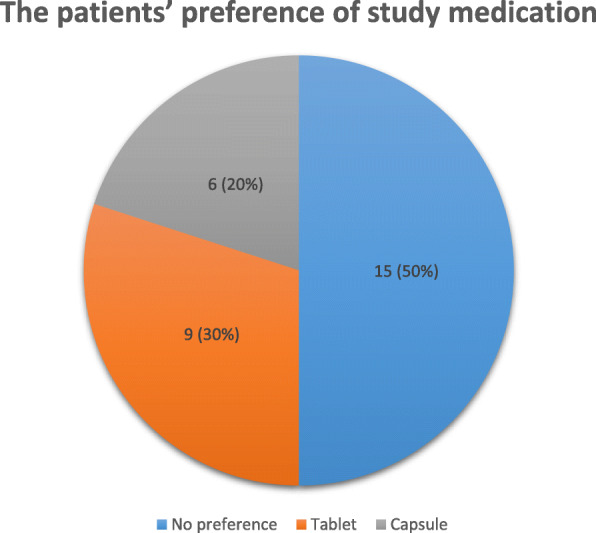
The patients’ preference of study medication**.** Fifteen patients (15/30) did not express a preference for one of the formulations but 30% (9/30) preferred UDCA tablets and 20% (6/30) capsule

Patient preference for the study formulation (tablets or capsule) was assessed at the last visit of study. Fifteen patients (50%) did not express a preference for one of the formulations but 30% (9/30) preferred UDCA tablets. The most important factor for the decision on the drug formulation was size, taste and packaging of the tablets and capsules (Fig. 2).

### Adverse event

**Fig. 3:**
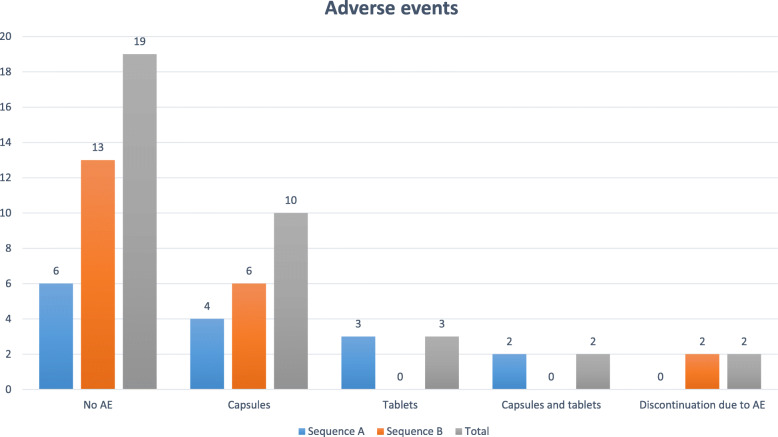
Adverse events. Most patients did not have AE 55.88% (19/34). The majority of adverse events were with capsule 29.41% (10/34) resulted more AE compared with tablets. No patient of sequence B had AE using UDCA tablets while sequence A had adverse event in UDCA tablets and capsules 5.88% (2/34). The discontinuation due to adverse event occurred in 5.88% (2/34) of patients in the sequence B. Legend: AE = adverse events

Most patients did not have an adverse event (AE) 55.88% (19/34). The majority of adverse events were with capsule 29.41% (10/34) resulted more AE compared with tablets. No patient of sequence B had AE using UDCA tablets while sequence A had adverse event in UDCA tablets and capsules 5.88% (2/34). The discontinuation due to adverse event occurred in 5.88% (2/34) of patients in the sequence B (Fig. 3).

## Discussion

This was a randomized controlled crossover trial of 30 patients with PBC that demonstrated no difference between UDCA tablets and capsules manufactured by the university hospital compared with standard UDCA. In addition, it showed a reduced treatment cost of 66.1% without significant biochemical worsening. It indicated that patients used the medication correctly or at least with the same regularity that were using before.

Hepatic parameters ALP, GGT, ALT, AST and total bilirubin were chosen in our study to assess therapeutic efficacy while Hopf et al. [13] used, ALP, GGT and ALT. Alkaline phosphatase was principal parameter because it is regarded as the most reliable surrogate marker for effective PBC treatment that correlates with survival [6, 14]. EASL [5] recommends that elevated serum bilirubin and ALP can be used as surrogate markers of outcome for patients with PBC, and routine biochemistry and hematology indices should underpin the clinical approaches to stratify individual risk of disease progression.

UDCA tablets formulations were tolerated better compared with capsules. Two patients had adverse event with capsule, they had severe diarrhea, which required reduction/fractionation of the dosage, but they did not improve (Table 4). These patients did not tolerate very well the capsule formulation and terminate the trial prematurely. Pharmaceutical formulations are mixtures of the pharmaceutically active ingredient and selected inactive ingredients. The added inactive ingredients (the excipients) might also help to keep the drug stable for a finite period [15]. UDCA tablets and capsules have difference formulations, so difference excipients and it may have the adverse event.

**Table 4:** Adverse events during the treatment period (*n* = 34). Description about adverse events during the treatment period in sequence A and B with UDCA 300 mg capsules and tablets manufactured by the university hospital during all 24 weeks

Adverse Event	Sequence A (*n* = 15) n (%)		Sequence B (*n* = 19) n (%)	
	Tablets	Capsules	Capsules	Tablets
Headache	0	0	1 (4)	0
Diarrhea	0	0	3 (12)	0
Abdominal pain	0	0	1 (4)	0
Flatulence	2 (8)	1 (4)	1 (4)	0
Acute myocardial infarction^a^	0	1 (4)	0	0
Urinary infection	0	0	1 (4)	0
Improvement in intestinal function	1 (4)	3 (12)	1 (4)	0
Nausea	0	0	1 (4)	0
Worsening of rosacea	1 (4)	1 (4)	0	0
Itch	3 (12)	1 (4)	1 (4)	0
Vomit	0	0	1 (4)	0

^a^ Occurred at the end of the study. Did not have it causal relationship established with the UDCA

One secondary endpoint assessed patients’ preference of UDCA formulations. It could be shown that half reported no preference between tablets or capsules but 30% patients preferred tablets supported the conclusion of adverse event. Hopf et al. [13] showed that many patients preferred tablets (45.3%) compared to only 15.6% of patients preferring capsules.

In Brazil we just have standard UDCA tablets and UDCA capsules in Europe [16]. So hospital pharmacy manufactured two different formulations, UDCA 300 mg tablets and capsules but tablets would be more advantageous for the hospital because it cost is lower than to the capsule, $6.91 and $7.02, respectively. In addition, UDCA capsules manufactured involved a manual process that depends on trained people to do and capsules took long production lead time compared with UDCA tablets.

In a prospective study, Marin et al. [4] demonstrated that the costs of production were assessed and compared with standard drugs prices indicating savings of 63.5%. Our study showed similar results with 66.1% when compared with purchase price for a public hospital. The observed savings, allied with the convenience and reliability with which the pharmacy performed its obligations, support the contention that internal manufacture of pharmaceutical formulations was a cost-effective alternative.

This study has limitations. Firstly, few patients were randomized and we did not get patients enough because PBC is an important but uncommon disease [5]. In order to draw more solid conclusions about manufactured drug, we should consider carrying out multicenter studies, including more patients and a control group. Secondly, disadvantage of a crossover design is that carryover effects may be confounded with direct treatment effects, in the sense that these effects cannot be estimated separately because it did not have a washout period that could reduce the impact of carryover effects. In addition, these carryover effects yield statistical bias [11].

UDCA tablets and capsules manufactured showed to be a viable alternative. In this way, it did not depend on the logistics of standard UDCA, decreasing lack of access and therapeutic failures. It provided a reduction in hospital costs compared with standard drugs available. This study represented a social and economic importance in the financial sustainability of public resources.

## Conclusions

In conclusion, this study demonstrated no difference on biochemical results between UDCA tablets and capsules manufactured by the university hospital compared with standard UDCA. Half patients reported no preference between tablets or capsules. However processing UDCA tablets is cheaper (reduction of 66.1% PBC treatment costs) and the preferred formula among the patients with less adverse event.

## Data Availability

The datasets used and/or analysed during the current study available from the corresponding author on reasonable request.

## Abbreviations

ALP: Alkaline phosphatase
ALT: Alanine aminotransferase
AST: Aspartate aminotransferase
GGT: Gamma glutamyl transferase
TBIL: Total bilirubin
AMA: Antimitochondrial antibodies
PBC: Primary biliary cirrhosis/cholangitis
UDCA: Ursodeoxycholic acid
ULN: Upper limits normal
AE: Adverse event

## References

[1] Jessup RL, O'Connor DA, Putrik P, Rischin K, Nezon J, Cyril S (2019). Alternative service models for delivery of healthcare services in high-income countries: a scoping review of systematic reviews. BMJ Open.

[2] Ribeiro E, Crozara MA, Nita ME, Secoli SR, MRC N, SK ON, ACC C, Santi FM (2010). Farmacoeconomia aplicada ao Hospital. Avaliação de tecnologias em saúde: evidência clínica, análise econômica e análise de decisão.

[3] Packeiser PB, Gindri RD (2014). Pharmacoeconomics: a tool for the management of drugs expenditures in public hospitals. Infarma: Ciências Farmacêuticas.

[4] Marin ML, Chaves CE, Zanini AC, Faintuch J, Faintuch D, Cipriano SL (2001). Cost of drugs manufactured by the university hospital role of the central pharmacy. Rev Hosp Clin Fac Med Sao Paulo.

[5] Hirschfield GM, Beuers U, Corpechot C, Invernizzi P, Jones D, Marzioni M (2017). EASL clinical practice guidelines: the diagnosis and management of patients with primary biliary cholangitis. J Hepatol.

[6] Lindor KD, Bowlus CL, Boyer J, Levy C, Mayo M (2018). Primary biliary cholangitis: 2018 practice guidance from the American Association for the Study of Liver Diseases. Hepatology..

[7] Poupon R, Lindor KD, Robson KM. *UpToDate*. Overview of the treatment of primary biliary cholangitis (primary biliary cirrhosis). 2018. https://www.uptodate.com/contents/overview-of-the-treatment-of-primary-biliary cholangitis-primary biliarycirrhosis? search=primary%20 biliary%2 0cirrhosis&amp;source=search_result&amp;selectedTitle=1~145&amp;usage_type=default&amp;display_rank=1. Accessed 09 Aug 2018.

[8] Boonstra K, Beuers U, Ponsioen CY (2012). Epidemiology of primary sclerosing cholangitis and primary biliary cirrhosis: a systematic review. J Hepatol.

[9] Wellek S, Blettner M (2012). On the proper use of the crossover Design in Clinical Trials Part 18 of a series on evaluation of scientific publications. Deutsches Arzteblatt Int.

[10] Nolan SJ, Hambleton I, Dwan K (2016). The use and reporting of the cross-over study Design in Clinical Trials and Systematic Reviews: a systematic assessment. PLoS One.

[11] Steven P. Design and Analysis of Clinical Trials: Crossover Designs. The Pennsylvania State University; [Internet]. 2018. https://newonlinecourses.science.psu.edu/stat509/node /123/. Accessed 27 Feb 2019.

[12] Dwan K, Li T, Altman DG, Elbourne D (2019). CONSORT 2010 statement: extension to randomised crossover trials. BMJ.

[13] Hopf C, Grieshaber R, Hartmann H, Hinrichsen H, Eisold M, Cordes HJ (2013). Therapeutic equivalence of Ursodeoxycholic acid tablets and Ursodeoxycholic acid capsules for the treatment of primary biliary cirrhosis. Clin Pharmacol Drug Dev.

[14] Pares A, Caballeria L, Rodes J (2006). Excellent long-term survival in patients with primary biliary cirrhosis and biochemical response to ursodeoxycholic acid. Gastroenterology..

[15] Kulkarni V, Shaw C, Kulkarni V, Shaw C (2016). Introdution. Essential chemistry for formulators of semisolid and liquid dosages.

[16] Rodrigues JPO (2015). Ursacol: acid ursodeoxycholic [drug product insert - health professionals].

